# Treatment Responsivity in Adolescents With Disruptive Behavior Problems: Co-Creation of a Virtual Reality–Based Add-On Intervention

**DOI:** 10.2196/46592

**Published:** 2023-11-28

**Authors:** Renée E Klein Schaarsberg, Amber Z Ribberink, Babette Osinga, Levi van Dam, Ramón J L Lindauer, Arne Popma

**Affiliations:** 1 Child and Adolescent Psychiatry & Psychosocial Care Amsterdam UMC location Vrije Universiteit Amsterdam Amsterdam Netherlands; 2 Garage2020 Dutch innovation network for societal youth challenges Amsterdam Netherlands; 3 Mental Health Amsterdam Public Health Amsterdam Netherlands; 4 Department of Child Development and Education University of Amsterdam Amsterdam Netherlands; 5 Child and Adolescent Psychiatry Amsterdam UMC location University of Amsterdam Amsterdam Netherlands; 6 Levvel Academic Center for Child and Adolescent Psychiatry Amsterdam Netherlands

**Keywords:** virtual reality, role-playing, cognitive behavioral therapy, co-creation, disruptive behavior, mentalization, adolescence, mental health, child, youth, clinical practice, intervention

## Abstract

**Background:**

We developed Street Temptations (ST) as an add-on intervention to increase the treatment responsivity of adolescents with disruptive behavior problems. ST’s primary aim is to improve adolescents’ mentalizing abilities in order to help them engage in and benefit from psychotherapy. Additionally, virtual reality (VR) is used to work in a more visual, less verbal, fashion.

**Objective:**

By recapping the lessons learned while developing ST so far, we aim to design the following study on ST. Furthermore, we aim to enhance the development and study of new health care interventions in clinical practice, together with adolescents as their end users.

**Methods:**

We followed an iterative co-creation process to develop a prototype of ST, in collaboration with adolescents and professionals from a secured residential facility in Amsterdam, the Netherlands. The prototype was tested during a pilot phase, involving 2 test runs, in which 4 adolescents and 4 professionals participated. Qualitative data were collected through interviews with the adolescents and by conducting a group interview with the professionals, in order to gain first insights into ST’s usability, feasibility, and its added value to clinical practice. In between the first and second test runs, the prototype was enhanced. On the basis of the complete pilot phase, we reflected on the future development and implementation of ST to design a subsequent study.

**Results:**

Over the course of 6 months, ST’s first prototype was developed during multiple creative sessions. Included was the development of a short 360° VR video, to serve as a base for the mentalization exercises. The final version of ST consisted of 7 individual therapy sessions, incorporating both the VR video and a VR StreetView app. On the basis of the qualitative data collected during the pilot phase, we found preliminary signs of ST’s potential to support adolescents’ perspective-taking abilities specifically. Additionally, using VR to focus on real-life situations that adolescents encounter in their daily lives possibly helps to facilitate communication. However, several challenges and requests concerning the VR hardware and software and the implementation of ST emerged, pointing toward further development of ST as an add-on intervention. These challenges currently limit large-scale implementation, resulting in specific requirements regarding a subsequent study.

**Conclusions:**

In order to gather more extensive information to shape further development and study treatment effects, a small-scale and individually oriented research design seems currently more suitable than a more standard between-subjects design. Using the reflection on the lessons learned described in this report, a research protocol for a forthcoming study on ST has been developed. By presenting our co-creation journey thus far, we hope to be of inspiration for a more co-creative mindset and in that way contribute to the mutual reinforcement of science and clinical practice.

## Introduction

Adolescent disruptive behavior is often a persistent problem [1,2]. Such behavior can be characterized by irritable, disobedient, hostile, and aggressive behavior [3]. Disruptive behavior may predispose to multiple negative outcomes, such as school dropouts, substance use, and nonviolent and violent delinquency when left untreated [4-6]. Regarding treatment, cognitive behavioral therapy (CBT) is often recommended. CBT is one of the most substantially studied types of psychotherapy, and multiple meta-analyses support CBT as an effective form of treatment for adolescent disruptive behavior [7,8]. In addition to this individual therapy method, an increasing number of evidence-based systemic interventions are available as well [9].

Although effective treatment options regarding disruptive behavior problems exist, not all youths respond to these options [7]. Treatment responsivity indicators (eg, cognitive abilities, denial, or motivation) are strong predictors of not successfully completing treatment. When focused on, such indicators function as preconditions for potential responsiveness to effective treatment options. When developing and implementing interventions, low cognitive abilities, such as poor verbal skills, warrant specific attention [10]. In this instance, using visual representations might be beneficial.

Virtual reality (VR) offers innovative possibilities regarding visual representations. Due to its visual presentation, VR tends to reduce the demand for cognitive abilities needed for the creation of mental representations of (hypothetical) situations [11]. These representations are often needed for common CBT exercises, such as role-playing. Additionally, by using VR, a safe learning environment is offered as an alternative to unsafe or unethical situations that would otherwise have to be created to mimic real-life situations to practice with [12]. Creating this VR environment would enable treatment to come as close as possible to real-life social situations adolescents encounter in their daily lives.

Using VR to visualize such situations can be especially supportive with regard to practicing mentalization, another precondition to engage in and benefit from psychotherapy [13]. This mental process, by which behavior is interpreted on the basis of intentional mental states such as thoughts and feelings [13], allows people to understand and make sense of their social world [14]. Adolescents with disruptive behavior problems often experience difficulties with this reflective ability [15]. By using VR to create real-life social situations, realistic opportunities to practice mentalization can be generated.

To increase the treatment responsivity of adolescents with disruptive behavior problems, we started the co-creation of an add-on intervention: Street Temptations (ST). By incorporating elements of CBT and role-playing techniques in practical and dynamic exercises using VR, ST aims to improve adolescents’ mentalizing abilities while working in a more visual, less verbal, manner. By helping adolescents reflect on both their own behavior and that of others, we intend to improve adolescents’ ability to engage in, and benefit from, psychotherapy. The co-creation framework entails a collaboration between targeted end users, potential other stakeholders, and academic researchers [16]. Co-creation is thought to increase effectiveness and adherence because it specifically empowers end users to create solutions tailored to their needs and circumstances [17,18].

In this early report, we elaborate on ST’s development thus far. By recapping the lessons learned and reflecting on them, we aim to gain a better understanding of ST as a co-created intervention. As a result, we aim to develop a forthcoming study on ST, to gain further insights regarding the program and future development of it. Moreover, our purpose is to provide other health care researchers and developers with valuable and transferable lessons.

## Methods

### Co-Creation Framework

The developmental process of ST is based on the co-creation framework by Leask et al [19], who have described 4 steps regarding the co-creation of an intervention: planning, conducting, evaluating, and reporting. First, the process is planned by framing the aim and identifying the appropriate sample. Then, the development of the program is conducted, including an iterative process of pilot testing. After development, the effectiveness of the intervention can be evaluated by locally testing the effectiveness at first, followed by testing at the population level. In this paper, we focus on the lessons learned during the conducting phase, in order to explicitly incorporate this knowledge into the preparation of the evaluation phase.

### Prototype Development

The idea for ST arose after an exchange between Garage2020 and the Dutch army. Garage2020’s mission is developing and implementing innovative solutions regarding youth development issues. Among others, designers, researchers, and experts regarding youth development are part of their multidisciplinary team. During the exchange, the army demonstrated how they use VR for military battlefield training in a way that is both safe and realistic. This led to the question of whether VR could also be relevant for youth care, allowing adolescents to practice with challenging situations they encounter in their daily lives, in a safe and realistic way. ST’s concept was developed during a workshop facilitated by Garage2020, with 30 youth care professionals, adolescents, and police officers. After identifying the aim and the target population, prototype development started.

ST’s first prototype was developed in collaboration between Garage2020 and a secured residential facility from the youth care provider Levvel in Amsterdam, the Netherlands. The core team that developed the first prototype consisted of 5 people with different professional backgrounds (ie, content developer, researcher, senior therapist, child psychologist, and lived experience expert). This team facilitated multiple creative sessions over a period of approximately 6 months, to brainstorm about the prototype and gather input from adolescents, and other professionals and stakeholders. Adolescents varied in their willingness to participate from session to session. Consequently, there was no core team of adolescents involved. The problems exhibited by the adolescent population in the facility consist of externalizing problems, often accompanied by internalizing problems, sometimes in combination with psychiatric and addiction problems. Adolescents are mainly placed here on court order.

### Pilot Phase

The first prototype was tested and developed further during a pilot phase, together with the secured residential facility. To enhance further development of the prototype, 2 test runs were planned to make improvements after the first test run and directly enable feedback on a second version of the prototype. These improvements were based on informal and low-threshold information exchange, during which no prestructured data were collected.

At the end of the pilot phase, adolescents participated in semistructured interviews to more robustly gauge participants’ experiences regarding VR and their participation in ST. ST trainers and a clinician participated in a semistructured group interview to discuss the feasibility of using ST for future implementation and research purposes.

The conducted interviews were audiotaped and transcribed nonverbatim. Because of the specific reflection on the lessons learned during the conducting phase to design a subsequent study on ST, it was deemed necessary to deduce the most important and relevant results regarding the forthcoming process. Therefore, we did not conduct a thorough qualitative analysis of verbatim transcripts. Instead, we choose to roughly code the nonverbatim transcripts to deduce the most important results relevant to our current objectives. Key quotes were translated into English to illustrate the outcomes.

### Ethical Considerations

The Ethics Review Board of the University of Amsterdam reviewed and approved the study (2019-CDE-10237). Informed consent from participants—and their legal guardians if younger than 16 years—was required and obtained for all participants. Study data were collected anonymously or deidentified when anonymous data collection was not possible. No potentially identifiable human images or data are presented in this paper. Collected data were stored safely at the research department or on a protected hard disk driver. Participants were not compensated for their participation in the study.

## Results

### Prototype Development

#### Overview

**Figure 1:**
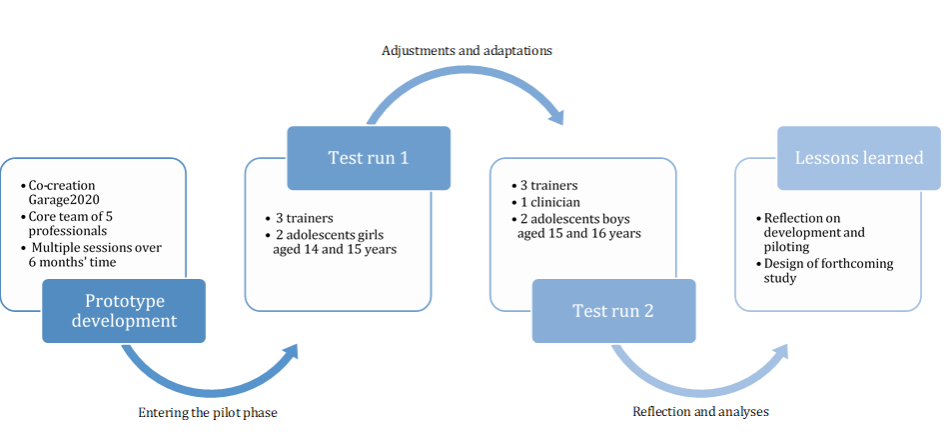
Overview of the co-creation process of Street Temptations.

A schematic overview of the complete process involving prototype development and piloting is shown in Figure 1. The creative sessions that were held during the first 6 months resulted in ST being an individual add-on intervention of four 45- to 60-minute meetings, based on one 360° VR video. The adolescent following the intervention will henceforth be called “adolescent.” The VR actors are referred to as “youngsters.”

#### The VR Video

The theme of the VR video was based on an important cause of adolescent disruptive behavior as discussed during the sessions, namely violence resulting from peer pressure. The video shows a situation in which youngster 1 forces youngster 2 to beat up youngster 3 when he passes by. Youngster 2 obeys, and in between the scenario, there are interview fragments shown in which youngster 2 is asked why he knocked down youngster 3. At the end, a compilation of videos from the internet of real fights between adolescents is shown, to emphasize the reality of the shown situation.

The video is watched from a third-person perspective, to create the perspective of a bystander and allow for evaluation of all other perspectives present. In total, the video takes approximately 3 minutes to watch. Adolescents watched the video at least 1 time per meeting. The HTC Vive VR headset (HTC Corporation) was used as hardware, together with the SteamVR software (Steam). Adolescents were allowed to either stand up or sit down while watching the video. No interaction in the VR video was possible.

#### The ST Meetings

ST’s first meeting serves as an intake session, in which adolescents formulate a learning goal. The 3 consecutive sessions revolve around the VR video described above. After watching the video, several analog exercises outside the VR environment follow to analyze the scenario from the perspectives of the 3 presented youngsters. In each session, one of these perspectives is highlighted. Together, the adolescent and trainer create a backstory for each character based on various building block cards, for example, “family” and “sports.” After giving a personal story to the character, the adolescent switches perspectives with the character. The trainer then challenges the adolescent to reflect on the scenario based on the specific character and the created backstory. After this reflection, the adolescent switches back to their personal perspective to discuss the differences and similarities between the 2 perspectives and why those might be present. A cognitive-behavioral approach is used to guide this analysis by focusing step by step on what the specific youngster thinks, feels, and does, together with role-playing elements to enhance perspective-taking. A set of playing cards was developed to visually support perspective-taking, as well as a workbook to support the exercises.

### Pilot Phase

#### Overview

During the pilot phase, 3 therapists were trained by the content developer from Garage2020. A clinician from the facility was added to the team after the first test run, to strengthen implementation and help identify adolescents who could participate. In total, 4 adolescents took part in testing the prototype.

#### First Test Run and Adjustments

A total of 2 girls (14 and 15 years old) participated in the first test run of the prototype. ST consisted of four 45- to 60-minute sessions at this time, with 1 session per week. Adolescents received the training from the same ST trainer each session. Based on the information exchange during and after the first test run, 1 adjustment and 2 extensions to the prototype were done.

The adjustment entailed stopping the use of a workbook. When showing adolescents the VR video, trainers noticed that it was difficult to motivate the adolescents to work with the workbook. Instead, it became clear that adolescents mainly wanted to start the conversation about the video and their own experiences. Because of this, a narrative approach was chosen for the exercises over a written approach using the workbook.

The first extension was the addition of a second module with 2 sessions. In this new module, the same analog exercises are used as in the first module, but rather than on the VR video, they are based on a real personal experience from the adolescents. The adolescents visualize this experience using a VR StreetView app. This app does not show the specific scenario each adolescent chooses to work with; instead, the adolescent is visually transported back to the exact place their experience took part. This visual transportation is used by the adolescent to explain aloud the specific situation that happened at that place. During the visualization, the VR glasses are streaming to an extra screen, allowing the trainer to watch along. By incorporating these personal situations, more attention is given to the transfer of what adolescents have learned during the first module, to their personal lives. This transfer was found to be not emphatically enough present within the first module, yet found essential to be added.

The second extension entailed the addition of a seventh meeting. This last meeting served as a reflection on the content and lessons learned during the meetings. Together with the first meeting, the intake, it was thought that this closing session would underscore the personal learning goal of the adolescent from start to finish.

#### Second Test Run and Overall Reflection

During the second test run, the new version of ST with 7 weekly sessions of 45-60 minutes was tested. The 3 trainers from the first test run participated again. A total of 2 boys (aged 15 and 16 years) participated in 4 of the 7 ST sessions. Trainers were again maintained consistent.

In the interviews, adolescents mentioned that they liked working with VR. Adolescents’ reactions to the VR video were for example: “It looks like I am there” and “It makes me think of a time when I was in a fight myself.” A lived experience expert, who took part in the core team, mentioned that a treatment setup like this could accelerate the treatment process: “VR takes me in six seconds to where I would otherwise be in six therapeutic sessions. I immediately experience all the feelings and thoughts that occur to me in such a situation, which makes me more open to discuss these.”

Regarding the ST intervention as a whole, 1 adolescent mentioned: “I thought it was informational, it was good to put yourself in another person’s shoes. I have learned to allow more feelings and thoughts.” Another stated: “If you know who the other person is, you are less likely to do something like that. Because you can think about how that person feels.” Another adolescent wanted to increase the number of sessions per week: “I would like to do it more days a week. ... I was working on building the characters and would like to continue with that.”

The trainers enjoyed the training they received to learn about working with ST. However, the content developer of Garage2020 who trained the trainers left after the first test run. The successor lacked the necessary background and knowledge to accurately supervise trainers. They mentioned: “The evaluation moments and supervision were missing” and “You really need supervision. Trainers talk to each other, but that is not enough.” This resulted in stagnation among the trainers. Trainers found this unfortunate because they were very enthusiastic about ST’s design and the idea behind the intervention.

During both test runs in the pilot phase, trainers worked with the HTC Vive VR headset (HTC Corporation). This headset requires a system of external sensors and a complex computer. This setup proved to be prone to technical difficulties. Trainers were not always able to get the VR material working: “That the computer sometimes did not work was frustrating.” After the replacement of the content developer, trainers lacked an accessible contact person who could help them encounter these technical issues. This caused a stagnation of sessions and a decrease in motivation. One trainer gave the following option: “Maybe the VR-headset should be wireless.”

Regarding further development of the intervention, all parties mentioned that it would be good to increase the amount of VR content. Trainers for example mentioned: “Every week they see the same video and additionally you have to do the same exercises for a few weeks. … That is too boring”, and “I think the product is fantastic, but it should have some more variety.” One of the adolescents thought: “Perhaps more stories should be added, to match better with what other participants might experience.”

## Discussion

### Reflection of What Was Learned

In this report, we reflect on the co-creation of ST as a VR-based intervention, focused on the enhancement of treatment responsivity indicators as preconditions for the effective treatment of adolescents with disruptive behavior problems. Results indicate the potential of ST as such, as adolescents, for example, explicitly mentioned positive effects regarding perspective-taking and working with VR. However, certain limitations regarding the intervention itself or its implementation arose as well, primarily acknowledged by the professionals. The associated implications for a subsequent study will be discussed in more detail.

Looking at the experiences regarding ST as a whole, an important finding is that it appears that adolescents preferred talking about the VR scenario, and sometimes even their own experiences, rather than a written approach. Along the same lines, Falconer et al [20] report findings on how a VR tool, albeit in a different form, seems to help facilitate communication during therapy. These are important indications, as adolescents can find it particularly difficult to engage in talking therapies such as CBT. Using VR might be helpful in facilitating this engagement and in that way possibly help to speed up the treatment process. These results fit well in the broader context of promising results that have been shown regarding VR-based interventions for mental health problems in children and adolescents [21].

Another important finding is that, although very preliminary, adolescents mentioned that ST helped them to look at different perspectives in a challenging social situation. Perspective-taking is an important component of mentalizing [22], ST’s primary target point. Previous studies have already shown, admittedly looking at different populations and working with other forms of VR, the positive effects of VR-based exercises regarding perspective-taking [23,24]. Considering that the population we currently focus on often experiences difficulties with mentalization [15], this result is promising and advocates for future research.

Considering the limited number of 4 adolescents who participated in the test runs, we have to acknowledge that sample size is likely going to be a difficult facet during the forthcoming process. Besides practical aspects that may hinder participation, lack of motivation is not uncommon within the larger population of adolescents within compulsory residential care [25-28]. Therefore, research designs requiring large samples with relatively meticulous inclusion and exclusion criteria are at this point likely to be less appropriate to study the effects of treatment.

With respect to the overall reflection after the second test run, trained ST therapists mentioned several important improvements regarding future implementation. Accurate supervision was 1 essential aspect. Supervision has indeed been shown to play an important role in the implementation of new programs and interventions [29]. The request for and importance of accurate supervision seems to argue for a small-scale study as well, to ensure that this need can be well met.

Improvements specifically aimed at the VR element were also mentioned. It is important that the hardware becomes more accessible to work with. Looking at the rapid development of VR hardware, this is currently a relatively simple improvement to make. Nowadays, all-in-one VR headsets such as the Meta Quests, requiring no external sensors or complex computers, are readily available. Using one of those all-in-one headsets in the future is therefore recommended, to simplify the use of the VR hardware.

In addition, adding more VR content is recommended. However, there are various ways in which VR content can be created [30]. The options range from relatively simple entry-level systems that deliver passive 360° video experiences, as currently used, to more high-end systems that enable interactive user engagement [12]. So far, mainly due to practical reasons, the type of VR software has not been an explicit topic of discussion in our trajectory. However, considering all the possibilities, different options will have to be weighed out against one another and several choices will have to be made accordingly.

Moreover, developing new content is expensive and time-consuming, meaning that this development needs to be well thought out. Alternatively, it is possible to use existing content. For example, there are VR cognitive behavioral therapy libraries, offering customizable VR environments. Nonetheless, such options are also not inexpensive and still need to be well thought out. Therefore, the future process will inevitably be subject to further development and changes. It will therefore be necessary to use a research design that will not collide with these developments and that is able to adapt to clinical practice within the requirements for good scientific research.

Taking the above reflections into account, several prerequisites for future research concerning ST arise. A subsequent study should be designed in such a way that a large and meticulous sample is not required, that accurate supervision for successful implementation can be ensured, and that the research will not collide with forthcoming developments and changes. When considering research designs that comply with these prerequisites, individualized study approaches appear to be a particularly good possibility for future research. Such studies, especially when using a single-case experimental design, can enable high-quality experimental research in a clinical setting, using repeated measurements with a small number of participants [31].

### Strengths and Limitations

The development of ST is embedded in a co-creation trajectory together with its end users. By explicitly reflecting on the development so far in this report, we can incorporate the lessons learned into further development and research. The total sample size of the pilot phase is a limitation in the developmental process so far, which therefore also influences this report. In addition, no adolescents have yet completed the entire program of ST. Consequently, results need to be interpreted with caution. However, the results provide preliminary insights into ST as a co-created intervention and provide valuable input for the forthcoming process.

### Conclusions

In conclusion, those involved mention preliminary signs of ST’s potential to innovatively support adolescents’ treatment responsivity. More specifically, perspective-taking, as an important aspect of mentalization, appears to be an interesting aspect to elaborate on, as well as using VR. However, there is room for improvement regarding several aspects, meaning that ST is not yet ready for large-scale implementation. Furthermore, the following research needs to be compatible with both the intervention and the circumstances under which the intervention is implemented and performed. To gather more extensive information to shape further development and study treatment effects, an individualized approach in which the effects of ST can be examined in detail seems most appropriate. Using the reflection on the lessons learned during the co-creation of ST thus far, the research protocol for the first effectiveness study on ST was developed. The paper on this protocol has been recently published [32]. By presenting our co-creation journey thus far, we hope to be of inspiration to more explicitly incorporate end users and their lived experiences into the development and evaluation of health care interventions.

## Abbreviations

CBT: cognitive-behavioral therapy
ST: Street Temptations
VR: virtual reality

## Data Availability

The data set generated during this study is not readily available because additional data potentially contains information that could compromise the privacy of research participants. The data can be made available by the corresponding author upon reasonable request.
